# Seeking Insights About Cycling Mood Disorders via Anonymized Search Logs

**DOI:** 10.2196/jmir.2664

**Published:** 2014-02-25

**Authors:** Elad Yom-Tov, Ryen W White, Eric Horvitz

**Affiliations:** ^1^Microsoft ResearchHerzeliyaIsrael; ^2^Microsoft ResearchRedmond, WAUnited States

**Keywords:** information retrieval, mood disorders, bipolar disorder, machine learning

## Abstract

**Background:**

Mood disorders affect a significant portion of the general population. Cycling mood disorders are characterized by intermittent episodes (or events) of the disease.

**Objective:**

Using anonymized Web search logs, we identify a population of people with significant interest in mood stabilizing drugs (MSD) and seek evidence of mood swings in this population.

**Methods:**

We extracted queries to the Microsoft Bing search engine made by 20,046 Web searchers over six months, separately explored searcher demographics using data from a large external panel of users, and sought supporting information from people with mood disorders via a survey. We analyzed changes in information needs over time relative to searches on MSD.

**Results:**

Queries for MSD focused on side effects and their relation to the disease. We found evidence of significant changes in search behavior and interests coinciding with days that MSD queries are made. These include large increases (>100%) in the access of nutrition information, commercial information, and adult materials. A survey of patients diagnosed with mood disorders provided evidence that repeated queries on MSD may come with exacerbations of mood disorder. A classifier predicting the occurrence of such queries one day before they are observed obtains strong performance (AUC=0.78).

**Conclusions:**

Observed patterns in search behavior align with known behaviors and those highlighted by survey respondents. These observations suggest that searchers showing intensive interest in MSD may be patients who have been prescribed these drugs. Given behavioral dynamics, we surmise that the days on which MSD queries are made may coincide with commencement of mania or depression. Although we do not have data on mood changes and whether users have been diagnosed with bipolar illness, we see evidence of cycling in people who show interest in MSD and further show that we can predict impending shifts in behavior and interest.

## Introduction

People in the United States and other developed countries spend a significant portion of their time online [1]. Anonymized logs of such activities provide an unprecedented opportunity for studies in public health as well as applications that serve individuals in a private manner, helping them monitor and improve the quality of their lives. One recent survey shows that 81% of people in the United States use the Internet [2]. When they seek medical information, 59% of those people search for it online. Web search engines play an important role in the provision of medical information to health consumers, especially because of the anonymity of online search [3], allowing people to comfortably seek out and review sensitive information.

Search logs gathered by commercial search engines such as Google, Bing, and Yahoo! enable privacy-sensitive analyses of people’s search behavior in the aggregate, across populations of users. The use of online resources for syndromic surveillance has been termed *infodemiology* [4]. Possibilities for leveraging signals from the Internet arise in the large-scale use of Web search for accessing health information. Prior research has studied multiple aspects of long-term search behavior in a medical context [5], for example, to identify flu outbreaks [6], improve medical retrieval [7], and to identify information needs of patients [8]. More generally, search log data has been used to study how people perform searches [9], in order to predict their next online actions [10,11], to predict their future interests [8], to improve search engines [12,13], and to understand in-world activities from long-term logs [5,14]

We present here analyses of the online behavior of people exhibiting an intense interest in mood stabilizing drugs (MSD), medications prescribed for helping patients with mood disorders. We find evidence that observed behaviors may be associated with episodes of mood swings and show that atypical periods of anomalous online behavior can be detected and predicted by observing past behaviors and comparing them to current observed behaviors.

Mood disorders (MD) are defined as a group of diagnoses in the Diagnostic and Statistical Manual of Mental Disorders (DSM IV) classification system relating to the changes in a person’s affective state. Mood disorders affect a significant percentage of the population, though varying ranges of incidence (9.3%-23.3%) have been reported [15,16]. The commonality of these diagnoses is that a disturbance in mood is the main underlying feature. Cycling mood disorders are characterized by intermittent episodes of the disease. Bipolar disorder (BD) is characterized by intermittent episodes of shifts in mood across a spectrum of affect, including periods of mania or hypomania. Such episodes degrade the quality of life for those afflicted. Therapy and medications are employed to limit mood-swing episodes. Medications include antidepressants, antipsychotics, and mood stabilizers.

Previous work [8] has shown that users who seek specific health information (eg, specific disease names) are largely patients and health care professionals. Thus, we shall assume that many of those searching for MD medications are those likely suffering from MDs, and we offer supporting evidence for this conjecture in the results presented later in the article. We analyze these searchers’ online behaviors and attempt to predict the onset and persistence of what appear to be episodes of significant swings in mood. We identify a candidate cohort by finding users with a high level of interest in specific mood stabilizing drugs (MSDs). Having identified these users and noted key aspects of their search behavior, we analyze a survey taken by people who have been diagnosed with a mood disorder. The data from the survey provides evidence that the searchers are more likely to be people suffering from mood disorders than they are health care professionals seeking information on patient care. The survey also provides evidence that shifts seen online (Dataset 1) with users’ interests in particular topics (eg, in adult material consumption) are correlated with episodes of mania. Finally, we show how changes in information needs of these users may indicate forthcoming mood swings. This work highlights the possibility of constructing applications that could operate in the privacy of patients’ own computing systems and serve to provide predictions about the likelihood of impending episodes. Such predictions might be used one day to guide preparations in advance of episodes or to gate interventions that might be used counter or minimize the level of debilitation associated with a mood swing.

## Methods

We extracted all English language queries submitted to the Microsoft Bing search engine by users in the United States for the six-month period from December 2011 to May 2012 (inclusive). We refer to this dataset as Dataset 1. For each query, we extracted the query text, time and date, a list of pages visited by the user as a result of the query, and an anonymized user identifier. An anonymized user identifier (a string hash) was generated and stored in a Web browser cookie on the user machine, enabling logging of Bing search queries and clicks on search results for computers over time. No other data were available via this method. We note two intrinsic limitations of the data used in the study: (1) we cannot distinguish between multiple users on the same machine, and (2) if a searcher uses the search service on multiple devices, they would appear in logs with separate identifiers, one per device.

In order to maintain user privacy, data were first anonymized by hashing, before the investigators had access to them. They were then aggregated prior to analysis and no individual-level user datum was examined by the experimenters. The Microsoft Research Ethics Advisory Committee reviewed and approved the methods and results, and provided insightful discussion and guidance on the study.

We defined queries on mood stabilizing drugs (MSD queries) as those containing the following specific drug names: Eskalith, Lithobid, Lithonate, Lithotabs, Valproic acid, Divalproex, Valproate, Depakote, or Depakene, as well as queries specifically mentioning the term “mood stabilizing” (and its derivatives) or Lithium (except where the term was used in conjunction with the term “battery”, “ion”, and similar terms).

A total of 127,803 users made such queries during the period of study. We note that the market share of Bing was reported to be approximately 16% during the data period (see, for example, [17]), and so the number of users querying for drugs is only a sample of the total population taking MSD.

In order to focus on people who are likely using MSD, we used a threshold of 5 MSD queries during the study period (similar to [8]) to identify users with a high level of interest in MSD. We identified 20,046 such users and extracted all queries posted by these users during the study period. On average, each of the latter users submitted 34 queries per week on all topics.

We used a proprietary classifier developed by the Microsoft Bing team to assign each query into a set of 63 categories, including, for example, commerce, tourism, video games, weather-related, and adult-themed queries. The classifier is used by Bing to determine whether to display special results such as instant answers. Queries could be classified into multiple categories (eg, purchase of flight tickets would be classified into both tourism and commerce).

To validate findings derived from analysis of the data drawn from Bing search, we performed identical analyses on behavioral data collected from an opt-in consumer panel recruited by Internet analytics company comScore. Millions of panelists provide comScore with explicit permission to passively measure all of their online activities using monitoring software installed on their computers. In exchange for joining the panel, participants are offered a variety of benefits, including computer security software, Internet data storage, virus scanning, and chances to win cash or prizes. In addition to logged search behavior, the comScore data also provides us with panelists’ gender and age (mostly bucketed in 5-year increments). Overall, the panelists were 53.04% (45,707/86,168) female, with the most prevalent age range being 25-34 years. We refer to the comScore data as Dataset 2.

Beyond the studies of online logs, we conducted a survey among 272 people who self-identified as being prescribed one of the drugs listed above. Respondents were recruited using the online survey website “Instant.ly”. The survey was comprised of 11 multiple-choice questions and 9 free-text questions. The survey is provided in Multimedia Appendix 1. We refer to the survey data as Dataset 3.

## Results

### Search Behaviors

A first set of results were generated on Dataset 1 via analysis of the searches from computers where the threshold number of 5 or more MSD queries were observed. We find that the pattern of observed searches changes significantly around the time that queries on MSD are issued. We provide evidence that such observed shifts in behavior are linked to the onset of mood swing episodes. After providing evidence that MSD queries can be used as a label for the onset of such events, we present a second set of results on the feasibility of predicting forthcoming episodes.

### Are Users Identified Through MSD Queries Likely to be Suffering From Mood Disorders?

Some users state the purpose of their query, for example, using queries such as “I have severe depression”. We counted the number of unique queries that mention a mood disorder (either using the term or using one of the terms “depression”, “mania/manic”, or “bipolar”) and whether or not they appeared in conjunction with the person to whom the mood disorder refers (either “I” or “my wife/ husband/ spouse/ son/ daughter/ boyfriend/ girlfriend”). Queries in the first person that mentioned mood disorders were 6.5 times (659 compared to 102) more likely than queries about mood referring to other people (statistically significant, *P*<.001, chi-square test). We take this as evidence that queries on mood disorders without reference to self or other tend to refer to the searcher.

Some of the above-mentioned drugs are also prescribed to people suffering from seizures or migraines. We found a ratio of 2.23 between the number of who people mentioned a term associated with mood disorders (the term itself or “depression”, “mania/manic”, or “bipolar”) and queried for MSD and the number of users who mentioned migraines or seizure and also queried about MSD drugs versus those who mentioned a term at the threshold numbers. This validates our focusing on users with mood disorders by analyzing the drugs prescribed for these disorders.

### Online Behavior Before and After MSD Queries

On average, queries on MSD were entered by searchers every 13 days (SD 11). While most users only rarely posted such queries, a sizable population of searchers posted MSD queries multiple times and on a large number of days during our study period. Users who made MSD queries, on average, made 17.6 queries per day on days when MSD queries were posted, compared to 8.7 queries (SD 16 for both) on days when they were not (statistically significant, *P*<.001, sign test).

For 98.42% (14,410/14,641) of MSD queries mentioning a specific drug name, a single drug was mentioned. A single drug was queried during the study period by 70.31% (5010/7126) of users who mentioned a specific drug name. Even among users who posted 10 or more queries containing an MSD name, 97.9% (860/878) mentioned a single drug name. Thus, user interest is predominantly in a single drug, even when interest in the drug is high.

**Figure 1:**
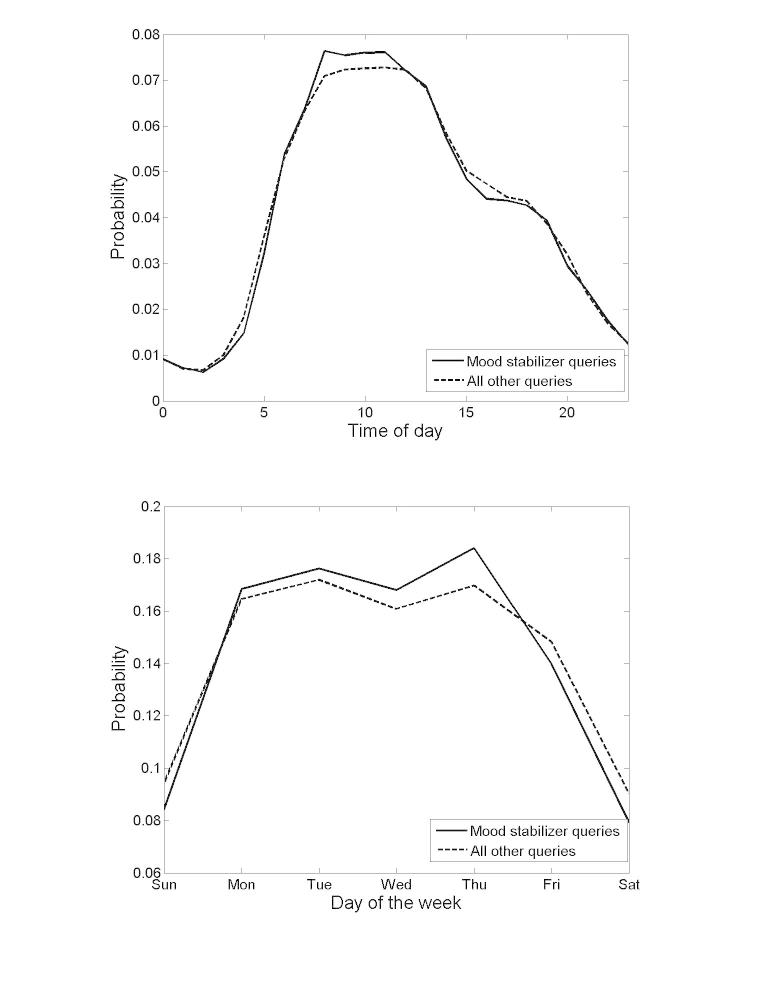
Probability of posting MSD queries as a function of time of day (above) and day of the week (below), compared to all other queries. This figure shows that MSD queries are more common in morning hours of weekdays.

Figure 1 shows the distribution of queries by time of day and by day of the week, partitioned by MSD queries and all other queries made by users in Dataset 1. As displayed in the figure, there is a slight increase in MSD queries during morning hours and on weekdays (vs weekends) (statistically significant, two-sample Kolmogorov-Smirnov, *P*<.001). This aligns with known MD patterns, where the most severe symptoms typically occur in the morning [18] and changes in daily routines are known to induce mood episodes [19].

**Figure 2:**
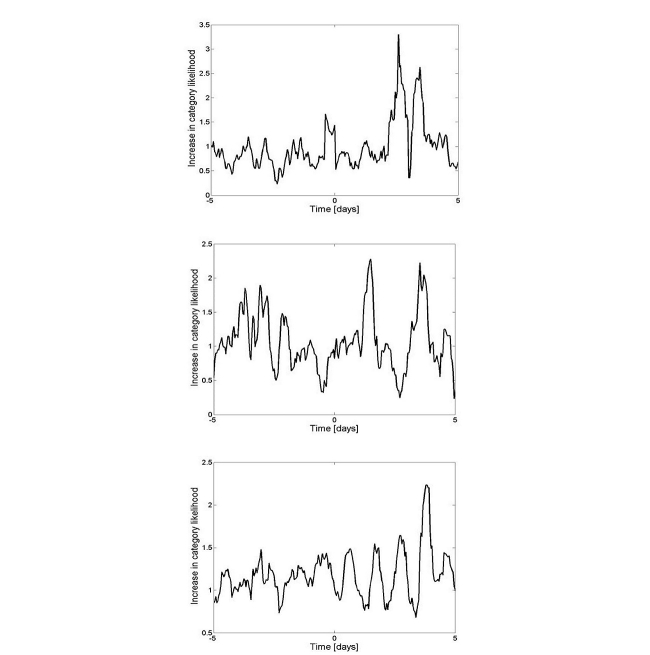
Change in query category likelihood as a function of time. Zero marks time of an MSD query. Categories represented are (from top to bottom): nutrition, business, and adult materials. The time series was smoothed with a moving average 5 hours in length.

Using the method described in [14], we synchronized the query stream of users to each day they posted an MSD query, and for each day measured the probability of posting queries in each of the query classes, compared to all queries. For the population studied, the categories of *nutrition, business,* and *adult* showed more than a doubling of the query probability as compared to all other query categories. Figure 2 shows the change in the likelihood of querying in these categories. The jump in query probabilities peaked at 3.30 for nutrition queries, 2.29 for business queries, and 2.24 for adult queries. We will show later that those who self-reported being afflicted with mood disorders reported similar increases in related topics when they experienced a mood swing. Comparing behavior before and after the time of the MSD query (marked as time zero), we note that all three categories show a large increase in likelihood on the day after the MSD query. This is especially noticeable in the adult queries, which show a diurnal cyclic behavior that increases in magnitude until four days after the MSD query. Interestingly, the peak in these queries is in the morning hours (8am-9am), corresponding to the peak of diurnal activity of MSD queries.

### Characterizing MSD (Mood Stabilizing Drug) Queries

The most common terms in MSD queries, excluding stop words and the drug names themselves, were manually partitioned by us into four mutually exclusive categories (with percentages of users mentioning terms in category in parentheses; see Table 1.

One of the most common concerns of people entering MSD queries are medication side effects. This is most noticeable when observing people who post MSD queries on at least 5 days of the study period. For these users, adverse drug reactions are an ongoing concern and the most common terms were “side effects” (both as a term pair and each word separately).

**Table 1:** Common MSD query terms.

Search term category	% of users mentioning terms (n=20,046) n (%)	Terms
Side effects	5391 (26.89%)	mood, effects, side, side effects, weight, effect
Drug-related	1847 (9.21%)	mg, long, take, dosage, dose, generic, release, interaction, gain
Disease-related	1516 (7.56% )	cause, bipolar, depression, high, low, treatment, anxiety, symptoms
Other	2467 (12.31%)	drug, blood, levels, children, use, sod, medication, code, sprinkles, used, taking, loss, time, list, normal, test, work, help, liver, patient, pain, lab, together

### Characterizing Clicked Pages

The informational goals of people querying on terms used to refer to MSD may be ambiguous. For example, a query for “lithium” could refer to the drug or the metal. To address such ambiguity, we analyzed the search results that users had clicked on using the click logs from the Bing search engine over the period of the study (Dataset 1). Queries followed by clicking on relevant links that are titled and summarized with text snippets are less likely to be ambiguous.

With MSD queries, we count the number of users and clicks that lead to each Web domain such as drugs.com. We remove effects of multiple clicks (eg, which may reflect user learning during the session) by analyzing the first result clicks for each query only. To remove noise from the click data, we focus only on clicks followed by a long dwell (30 or more seconds) on the landing page. Long dwells have been shown to correlate with satisfaction in prior studies of information-seeking behavior [20]. Table 2 lists the top 10 most popular domains, ranked in descending order by the number of users.

The results reveal that the top 10 clicked domains are health-related with the exception of wikipedia.org, answers.yahoo.com, and wiki.answers.com, which are comprised of reference information and social question answering data. We note that the average number of clicks (considering one click per session) per user is greater than 1, indicating that people visit these sites multiple times. We note that some sites (lower in the list) are unrelated to MSD and are linked to alternate meanings of “lithium” (for example, a channel on satellite radio, a song by Nirvana, a fitness training website, batteries, and mining companies). Given the prevalence and ambiguity of “lithium”, we removed or isolated users who queried only for this term in our later analysis.

**Table 2:** Top 10 clicked URLs following MD medication queries.

Clicked domain	No. of users	No. of clicks	Avg. no. clicks / user
drugs.com	5688	9989	1.756
en.wikipedia.org	4711	7136	1.515
ehow.com	2493	3252	1.304
wiki.answers.com	2270	3607	1.589
answers.yahoo.com	2199	3024	1.375
bipolar-disorder.emedtv.com	1786	2159	1.209
webmd.com	1529	1853	1.212
ncbi.nlm.nih.gov	1326	1827	1.378
healthcentral.com	1294	1540	1.190
bipolar.about.com	1237	1489	1.204

### Searcher Demographics

We next analyzed search logs purchased from comScore (Dataset 2), comprising user search behavior from comScore panelists over a 12-month timespan that overlapped with the period of time covering Dataset 1. Unlike Dataset 1, the comScore logs provide demographic information about searchers. The comScore data includes searches issued to all major Web search engines (Google, Bing, and Yahoo!), providing a broader user sample than the set of Bing users in Dataset 1. We also examined users who queried for at least one of the specific drug names mentioned above. In these data, users were tracked using an anonymous identifier connected to each individual panelist rather than with a machine-based identifier as in Dataset 1, which does not allow us to discriminate among multiple users of a single machine.

**Figure 3:**
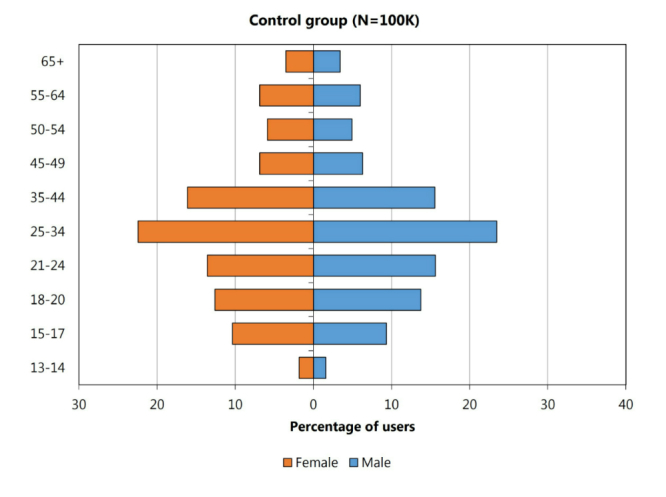
Age and gender distribution across a control group of 100,000 users.

**Figure 4:**
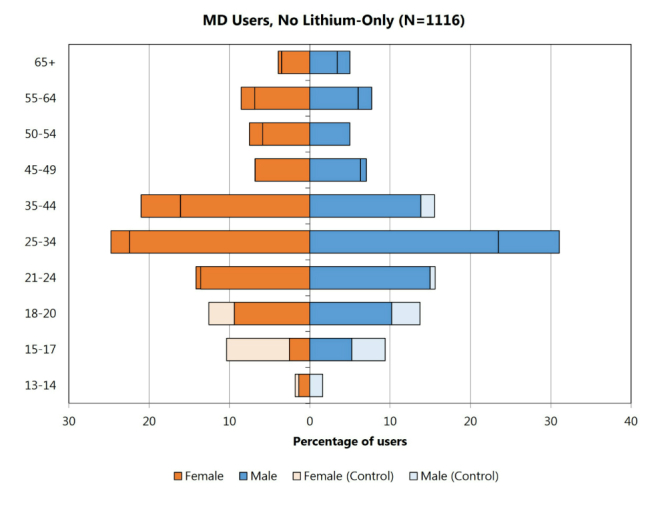
Age/gender distribution of users who searched for mood disorder medications, minus users who only searched for lithium. MSD plot is overlaid on the control plot from Figure 3. When the control value is larger, the control bars (in lighter shades) are visible. When the MSD value is larger, the MSD bars occlude the controls and are marked with lines in the MSD bar.

Beyond moving from potentially multi-user machines to individual searchers, the comScore data also provides searchers’ age ranges and gender. We sought to understand whether the distribution of searchers exhibited demographics that match those of known MSD patients. The dataset includes 1116 users who queried for MSD medications. A tornado diagram covering age and gender for a control group of 100,000 randomly chosen panelists is shown in Figure 3. Another tornado diagram displayed in Figure 4 shows the distribution of age and gender for users who query for MSD drugs. In comparing the test and the control group, we can see that women are more likely than men to query for MSD medications (control: 53.04%, 45,707/86,168, female vs MD users: 57.06%, 586/1027, female, statistically significant, two-proportion *z* test, *P*=.005). Although prevalence of bipolar disorder is similar across genders, the difference may reflect the fact that women are more likely to experience more severe effects and bipolar II disorder than men [21]. We also note a spike in queries on MSD, in the 25-34 age range for men and women, which is higher for men (statistically significant, chi-square test, *P*<.001). This aligns well with studies of mood disorders, which have shown that the median age for the development of mood disorders is 30 years [22]. These results provide evidence that the user group observed searching for MSD medications online may be similar demographically to the subset of the population known to be affected by mood disorders.

### Survey Data

To more fully understand the search behavior observed in the logs in Datasets 1 and 2, we conducted a complementary survey of 272 people who had self-identified as being prescribed one of the MSD listed above (Dataset 3). People in the survey cohort had a median age of 36 years (min: 18, max: 77), 25.4%, 56/220, were male, and had taken an MSD for an average of 3.2 years. Respondents reported having an average of 10.5 episodes per year, lasting an average of 5.5 hours. Approximately 97.7% (256/262) of respondents were prescribed a regular dose of the drug, 71.7% (188/262) daily, 17.9% (47/262) more than once daily, and the rest at less than daily doses), and 83.6% (214/256) reported that they comply with their prescription.

Only 18.3% (48/262) of respondents never searched for information on their MSD on the Internet; 30.9% (81/262) reported searching once in the past 6 months, 44.6% (117/262) between twice and 10 times, and 6.1% (16/262) reported searching more than 10 times. Thus, approximately 51.9% (133/262) of respondents made repeated queries about their medications. The most common triggers for searching were the first time that the drug was prescribed (71.8%, 188/262), when side effects occur (42.7%, 112/262), when the respondent felt the drug was not working (32.4%, 85/262), following discussions with friends or family (29.4%, 77/262), and at the outset of an episode (25.2%, 66/262).

Respondents sought information on side effects (82.1%, 215/262), efficacy (62.2%, 163/262), dosage (40.5%, 106/262), and retail locations for acquiring the medication (13.0%, 34/262). They found information at reference websites such as Wikipedia (55.3%, 145/262), consumer-oriented sites (47.3%, 124/262), social media (40.6%, 106/262), and information posted by drug manufacturers (38.2%, 100/262). These findings, particularly on the frequency searches for side effect and dosage information and the types of resources selected, align well with the log analysis performed on Dataset 1.

People who searched for information multiple times reported doing so because they needed more information (42.7%, 79/185), wanted reassurance that the drug was the right one for them (27.6%, 51/185), needed different information than before (18.9%, 35/185), or because they had forgotten information they had once known (10.2%, 19/185).

Approximately half (48.6%, 70/144) of the respondents reported that they changed their online search and browsing behavior when they experience a manic (“high”) state, and a similar percentage (55.9%, 79/179) reported a change in behavior during depression (a “low”). Of the respondents that provided input, 8.5% (41/144) mentioned doing unnecessary online shopping during manic states, 22.2% (32/144) reported researching new topics on the Web, 13.9% (20/144) look for information that will make them happy (including their hobbies), 13.9% (20/144) reported being more active on the Web in general, 8.3% (12/144) search for health information, and 6.2% (9/144) reported an elevated interest in searching sites with pornographic content.

Respondents reported that, during depression periods, they usually stay offline (68.2%, 122/179), a finding that is in line with those reported by De Choudhury et al [23]. Others reported looking for depressing topics (15.1%, 27/179) or shopping, so as to feel bad (2.8%, 5/179), which resonates with their depression. Others looked for health information (7.3%, 13/179) or sites that could make them happy (6.1%, 11/179).

### Predicting Queries via Users’ Query Streams

We posit that most of the searchers posting MSD queries above the threshold rate are actual patients taking MSDs and that the MSD information seeking is likely performed near the commencement of an MD event, possibly the start of a manic episode. The former is evidenced by the temporal patterns associated with such postings (ie, days of the week and time of day), the fact that most users were interested in a single drug, and the demographics of searchers. The latter is apparent from the behavioral changes associated with days before and after MSD queries are posted. For example, we see a jump in business-related (eg, shopping) queries (see Figure 2), an activity that was identified in the survey as correlated with manias. Moreover, a quarter of survey respondents reported searching for MSD when they feel that an episode is commencing, which suggests that some proportion of recurrent MSD queries are triggered by the onset of a mood swing. We believe that most MSD queries are related to manic events because, as reported in the survey, people who experience depression tend to stay offline.

Given that the days on which MSD queries are posted may be significant, we focus on the prediction of days on which MSD queries will be posted. We investigated three distinct populations of users: (1) Recurring MSD, (2) Occasional MSD, and (3) Lithium Only. Recurring MSD included users who posted MSD queries on at least 5 days during the data period. There were 498 users in this population. Occasional MSD were users who posted an MSD query that mentioned a specific MSD drug in a query, but did so on fewer than 5 days. There were 9633 users in this population. Lithium Only users posted a query that mentioned Lithium, but not a specific drug, and did so on fewer than 5 days. There were 9884 users in this population.

We represented the users’ daily query streams as vectors of the attributes detailed in Table 3. We augmented the daily activity vector of a user by concatenating the vector with the average vector of activity for up to the last 14 days (ie, the average of the past two days, the average of the past three days, etc.) Also, for each attribute in Table 3, we computed the difference between the attribute (eg, the number of queries in category) and average value of that attribute in all previous non-MSD days. These attributes represent divergence between typical and current activity. Finally, the day of the week was added as an attribute. Thus, a total of 1981 attributes (14 lags + 1 divergence, times 132 attributes, and one day of the week attribute) were used to represent the daily activity of each user.

We used the feature representation of the user to predict whether the user would post at least one MSD query in the following day. A separate classifier was trained for each user population. In order to obtain a valid comparison among populations, we chose a random subset of the Occasional MSD and Lithium Only populations of the same size as the Recurring MSD population (so as to afford similar sized training datasets), and report results on those subsets.

We constructed a decision tree [24] as a classifier. Five-fold cross-validation [24] at the level of searchers (to avoid problems of training on future data) was used to train and test the classifier; each user was randomly assigned to one of the five cross-validation folds. Classifier performance was measured by identifying the area under the receiver operating characteristic curve (AUC).

**Figure 5:**
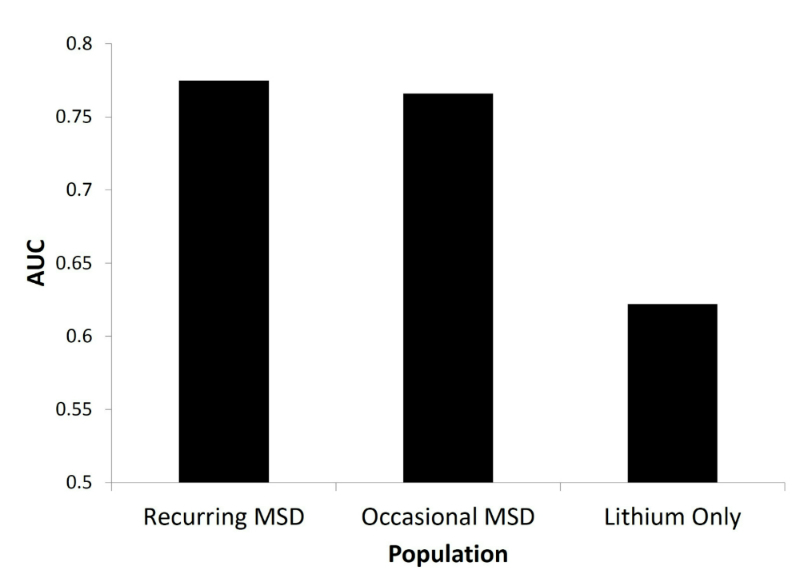
Area under the ROC (AUC) for the three populations of users.

The AUC for the three populations is shown in Figure 5. The highest AUC is obtained for the Recurring MSD population and the lowest for the Lithium Only population. The difference between the two MSD populations is not statistically significant, but differences are significant between the Lithium Only population and the MSD populations (*P*<.001) [25]. The findings could be attributed to differences in the number of samples per user or to the number of positive labels, although no statistically significant correlation was found. It is likely that the differences are in some way connected to inherent differences among the user populations.

The attributes selected more than twice among the 50 highest levels of the decision trees are listed in Table 4. First, we note that the number of attributes chosen for the MSD populations are much fewer than those selected for the Lithium Only population, suggesting that the first two populations are more homogeneous than the latter population. Second, we note the prominent appearance of adult-related queries in the Recurring MSD population, which is likely related to the activities shown in Figure 2. We also note that many of the attributes appearing in the MSD populations (and to a lesser extent, the Lithium Only population) are related to hobbies, a fact underscored by the reported interests of survey participants.

In view of the observed spike in adult-related queries following MSD queries, we evaluated the use of adult-related queries as an outcome and label and sought to construct predictive models to forecast whether a searcher would ask an adult-related query on the next day. We focused on a population that (as in the Recurring MSD population) asked an adult-related query in at least 5 days of the study period. There were 275 such users, 5.8% (16/275) of which also appeared in the Recurring MSD population and 77.1% (212/275) in the Occasional MSD population. The AUC for this prediction task was 0.71 (compared to 0.78 for MSD queries), suggesting that adult material is a strong proxy for behavioral changes followed by mood stabilizing events, an effect noted previously in several studies [26,27].

**Table 3:** Attributes of classifier for predicting days when mood stabilizing drug (MSD) queries are posted.^a^

Attribute	Number of attributes
Total number of queries per day	1
Total number of query topics per day, as represented by categories identified by query classifier.	1
Maximal number of queries per hour	1
Number of active hours per day	1
Number and fraction of queries posted in unusual hours (defined as 11pm to 4am local time).	2
Number of queries from each category (both in raw scores and after thresholding)	126

^a^The attributes are computed for each of 14 time lags, as well as the divergence from non-MSD activity, for a total of 1980 attributes.

**Table 4:** Most frequently selected attributes for each class of users.

User population	Features
**Frequent MSD** ^a^	
	Number of adult queries
	Divergence in the number of adult queries
	Number of vehicle-related queries
	Number of commerce-related queries
	Divergence in the number of commerce queries
	Number of event-related queries
	Divergence in the number of queries related to flight status
**Occasional MSD**	
	Day of the week
	Number of vehicle-related queries
	Number of book-related queries
	Divergence in the number of commerce queries
	Divergence in the number of celebrity-related queries
	Number of queries related to clothes and shoes
	Number of commerce-related queries
	Divergence in the number of commerce queries
**Lithium Only**	
	Day of the week
	Number of vehicle-related queries
	Divergence in the number of vehicle-related queries
	Number of book-related queries
	Divergence in the number of book-related queries
	Number of commerce queries
	Number of queries related to clothes and shoes
	Divergence in the number of commerce queries
	Divergence in the number consumer electronics queries
	Number of event-related queries
	Divergence in the number of finance-related queries
	Number of queries related to flight status
	Number of health-related queries
	Divergence in the number of health-related queries

^a^MSD: mood stabilizing drug

## Discussion

### Principal Findings

Analyses of logs of search activity show potential as a valuable tool in public health as well as for privately fielded applications and services that work on behalf of users. We identified a population of users who show a strong interest in MSD and also show significant changes in their online search behavior around the time of expressing interest in the medication. We showed that we can build predictive models that can be used to forecast the future appearance of MSD-centric search queries, which may be associated with the onset of a bipolar episode. We believe the possibility of predicting mood swing episodes with applications running within the privacy of a user’s own computing device might one day help patients and caregivers to better understand and prepare for impending changes in mood.

Comparing the results of the survey with the behavior observed online, we find several similarities. First, the ratio of the number of people who made between 2 and 10 queries and those who made more than 10 queries compared to those who made a single query is 1.4 and 0.2 in the survey, respectively, compared to 0.5 and 0.1 in the query log data. Second, the topics of search identified by respondents closely match those of the frequently occurring terms on MSD queries. Finally, respondents reported searching for information related to shopping, hobbies, and health information, as well as heightened search on adult content, which correspond to our observations on the online behavioral dynamics coinciding with users seeking information about MSD.

The appearance of repeated queries for MSD is an intriguing phenomenon. Survey respondents reported re-submitting queries on MSDs because they needed more or different information, or because they needed reassurance that the prescribed drug was the right one for them. Web data suggests that the latter rationale, together with concerns about side effects, were major causes for such repeat searches. We posit that the onset of a mood swing episode causes patients to become more aware of their disease in general and of their drugs in particular (especially when they are non-compliant), triggering an MSD search. Thus, such triggering behavior may not be limited to mood disorders, but to a wider class of diseases.

### Limitations

A key limitation of our study is the lack of a gold standard on outcomes. We cannot directly link users in our cohort with their real-life persona, and thus cannot know if they are, in fact, suffering from a bipolar mood disorder. To address this shortcoming, we note that previous work [8] has shown that seeking specific health information is done mostly by patients and primary caregivers. Second, the observed change in online behavior around the time of MSD queries provides evidence that users are experiencing a major change in their activities during this period of time. Third, the temporal appearance of MSD queries (at specific times and dates) and the demographic profile of these searchers are consistent with actual patients taking MSDs. Finally, users self-reported in a survey that they make similar changes in their online behavior. The most discriminatory features in our predictive models were those that quantified the change in behavior in several specific areas, including health and commerce. These topic areas were independently identified by survey respondents (in response to free-text questions) as topics that receive attention during manic periods. These findings provide evidence in support of our association of MSD queries with mood swing episodes and provides evidence that we are observing users who experience these events, especially of the mania type.

### Conclusions

Although drugs and behavioral treatment can reduce the incidence of mood disorder events, compliance with MSD prescriptions has been estimated to be as low as 35% [28]. The low compliance may be based on the side effects of these drugs [29]. However, non-compliance is associated with greater severity of mania events [30]. Predicting MD events before they occur may help people to better understand and prepare for changes in mood. We believe our study sets the stage for improved health and well-being for patients with mood disorders.

Opportunities for future work include working with patients to link online activities with clinical observations. Such efforts could validate our results and ascertain the accuracy of early warnings, as well as verify whether timely forecasts about an impending episode could be harnessed in beneficial ways.

## Multimedia Appendix 1

Survey of users taking MSDs.

## Abbreviations

AUC: area under the receiver operating characteristic curve
MD: mood disorder
MSD: mood stabilizing drug
