# Supplementary material for: Seeking Insights About Cycling Mood Disorders via Anonymized Search Logs
Source: J Med Internet Res. 2014 Feb 25;16(2):e65. doi: 10.2196/jmir.2664 (PMC3961703; doi:10.2196/jmir.2664)
Supplement: Supplementary file 1 [file jmir_v16i2e65_app1.pdf]

Appendix: Survey of users taking MSDs

Microsoft Research Online Health Search Study (Private and anonymous)

We are doing a study of peoples' experiences with fluctuating moods. This survey is designed to measure the kinds of information on the Web sought by people who have been prescribed mood stabilizing drugs.

The survey is designed to take around 5-10 minutes to complete. It is completely anonymous.

This survey is conducted by Microsoft Research, and will only be used for academic research purposes.

Please answer this survey if you have been prescribed a medication for bipolar disorder or other mood swings, including the following drugs: Eskalith, Lithiobid, Lithonate, Lithotabs, Valproic acid, Divalproex, Valproate, Depakote, or Depakene.

Question: Have you have been prescribed one of the following drugs: Eskalith, Lithiobid, Lithonate, Lithotabs, Valproic acid, Divalproex, Valproate, Depakote, or Depakene?

Answers: Yes \ No

Only users who answered “Yes” to this questions saw the following questions:

1. Question: My physician has prescribed me to take a regular dose (e.g. daily) of a mood stabilizing medication:

   Answer (choose one of): Yes \ No

2. If the answer to the previous question was "yes", do you comply with this prescription?

   Answers (choose one of): Yes \ Sometimes \ No \ My answer to the previous question was “No”

3. How often do you take your mood stabilizing medicine?

   Answers (choose one of): Several times a day \ Daily \ Weekly \ Occasionally \ Never

4. What is your prescribed dose (in mg) of the medicine?

   Answer: (open question)

5. How long have you been taking this medicine?

   Answer: (open question)

6. How many episodes do you have per year?

   Answer: (open question)

7. What is the typical duration of each episode?

   Answer: (open question)

8. How would you characterize the episode?

   Answer: (open question)

9. Do you think or know that you have side-effects from the medication?

   Answers (choose one of): Yes \ No

10. In the past 6 months, how many times (approximately) have you searched for information about this drug (or related drugs) using the internet?

    Answer (choose one of): Never \ Once \ 2-10 times \ More than 10 times

11. When did you search for information on the medication? Please select all appropriate answers

    Answer (choose one or more of): When it was first prescribed to me \ Following a discussion about it with friends or family members \ When I order a new prescription \ When I feel that the medication is or isn't working for me \ When I think I have side effects of the medication \ When I think I'm starting to have an episode \ Others, please specify.

12. What kind of information have you sought?

    Answer (choose one or more of): Side effects \ Dosage \ Places to buy \ Effectiveness of the drug for my condition \ Others, please specify.

13. Where did you find the information you were looking for? Please select all appropriate answers

    Answer (choose one or more of): Discussion with (or by) others taking the drug \ Consumer-oriented information about the drug \ General information sources such as Wikipedia \ Drug manufacturers \ Others, please specify

14. If you searched for information on the drug at multiple times, please select the primary reason for doing so:

15. Answer (choose one or more of): I forgot the information \ I needed more information \ I needed different information than before \ I needed reassurance that the drug was right for me \ I didn’t search multiple times \ Other, please specify.

16. Are you ever concerned that new symptoms are caused by the mood-stabilizing medication?

    Answer (choose one of): Yes \ No

17. If so, how do you find out more about possible symptoms?

    Answer: (open question)

18. When you are having a high, do you search the Internet for different topics? If so, how do your interests and activities change?

    Answer: (open question)

19. When you are having a low, do you search the Internet for different topics? If so, how do your interests and activities change?"

    Answer: (open question)

20. We are grateful for any comments you may have. Please comment below on any of the questions.

    Answer: (open question)
